# A large-scale screening of hepatitis C among men who have sex with men in the community using saliva point-of-care testing

**DOI:** 10.3389/fpubh.2024.1478195

**Published:** 2024-12-09

**Authors:** Sonia Albertos, Francesc X. Majo, Rafael Esteban, Joan Colom, María Buti

**Affiliations:** ^1^Division of Gastroenterology and Hepatology, Hospital Residencia Sant Camil, Consorci Sanitari de l’Alt Penedès i Garraf (CSAPG), Barcelona, Spain; ^2^Catalunya Healthcare System, Generalitat de Catalunya, Prevenció, Control i Atenció al VIH, les ITS i les Hepatitis Víriques, Barcelona, Spain; ^3^Department of Internal Medicine, Hospital Quiron, Barcelona, Spain; ^4^Liver Unit, Internal Medicine Department, Hospital Universitari Vall d’Hebron, Barcelona, Spain; ^5^Universitat Autònoma de Barcelona, Barcelona, Spain; ^6^Centro de Investigación Biomédica en Red de Enfermedades Hepáticas y Digestivas (CIBERehd), Instituto de Salud Carlos III, Madrid, Spain

**Keywords:** hepatitis C virus, screening, hepatitis C seroprevalence, men who have sex with men, quick saliva test

## Abstract

**Aim:**

To assess the feasibility and acceptability of massive hepatitis C virus (HCV) testing in point of care on the street using quick tests, determine the characteristics of the population included, and the prevalence of HCV infection in this population.

**Methods:**

Cross-sectional community-based study including adult men who have sex with men (MSM) who attended the three most important LGTB+ events in Sitges (Catalonia, Spain) in 2022. Points of care were set up on tents on the street and attendees were offered voluntary anti-HCV antibody self-testing. Participants were informed of the study, provided consent, completed the test for identification of risk practices (TIRP), and took the test with the OraQuick® HCV test on a saliva sample (sensitivity: 97.8% [95% confidence interval (CI), 93.2–99.4%] and specificity: 100% [95% CI, 98.4–100%]; gold standard: IgG antibody test for HCV by immunoassay [serum]); participants with positive results were offered HCV virus testing with the Xpert HCV Fingerstick® on a blood drop.

**Results:**

A total of 1249 adults participated in the large-scale screening, of which 1197 (95.8%) were identified as MSM. The screening time was 39 participants/h. Four (0.32%) participants had positive anti-HCV results, all with undetectable HCV RNA levels. Participants’ median (IQR) age was 44 (35, 54) years; most were Europeans, and 13% reported being unaware of their serological HCV status. The mean (SD) TIRP score was 1.40 (1.44) (*n* = 1062), with 67.41% reporting some risk, and the self-perceived sexually transmitted disease score was 3.0 (2.82) (*n* = 969).

**Conclusion:**

The point-of-care strategy on the street using a quick oral self-test at massive MSM events is feasible, well-accepted, and quick, and may be a useful strategy to reach other populations at risk of HCV infection.

## 1 Introduction

Even though acute hepatitis C virus (HCV) infection may clear up spontaneously, most cases become chronic, resulting in disease manifestations ranging from mild disease to advanced fibrosis, including cirrhosis and hepatocellular carcinoma (1). According to the World Health Organization (WHO), 58 million people worldwide lived with chronic HCV infection in 2016 (2). HCV is a blood-borne virus most frequently transmitted through infected medical supplies and blood products and by the sharing of injection equipment among injected drug users (1). Even though sexual transmission is less common (3, 4), the serological prevalence of HCV infection (i.e., the presence of anti-HCV antibodies) among men who have sex with men (MSM), particularly among those coinfected with the Human Immunodeficiency Virus-1 (HIV-1), is higher than in the general population (4%; range 2.9–13.0%) (5).

Despite the low efficiency of sexual HCV transmission, outbreaks of HCV infection have been reported since 2000, emerging as a real problem among MSM (3, 6, 7). The incidence of HCV infection has increased since 2000, particularly among HIV-1-positive MSM and HIV-1-negative MSM using Pre-Exposure Prophylaxis (PrEP) (3, 8, 9). Furthermore, the use of recreational drugs during sex is common in these populations and frequently associated with sexual risk behaviors (10, 11).

A large proportion of HCV cases remain undetected, especially among individuals outside routine care, precluding access to treatment with direct-acting antiviral agents (DAAs), the cure for HCV (3, 12). Consequently, in addition to prevention and treatment, identifying unknown and untreated HCV cases among the most prevalent populations is fundamental for eliminating HCV infection (2, 4). Different screening strategies targeting at-risk populations have been assessed in different settings, such as primary care, pharmacies, emergency departments, and prisons, to identify unknown HCV infections (13–16). In addition to these small, limited initiatives, a population-based nationwide screening in Egypt tested 79.4% of the population, and in the US, screening is recommended for all adults aged 18–79 years (17, 18).

Studies assessing large-scale, community-based screening programs targeting at-risk communities are limited and necessary to detect HCV-infected individuals. This study aimed to assess the acceptability and feasibility of massive HCV screening among MSM attending MSM events in Sitges (Catalonia, Spain) at point-of-care points (i.e., tents) set up on the street, based on self-testing using saliva quick tests. The secondary objectives were to determine the characteristics of the population included and the prevalence of HCV infection in this population.

## 2 Methods

### 2.1 Study design and population

This cross-sectional community-based study included MSM who attended the most representative MSM parties in Sitges (Catalonia, Spain). Men ≥18 years who participated in the Bear Sitges Weekend (April 29th – May 1st, 2022), the Sitges Gay Pride (June 2022), and the International Bear Sitges Week (September 2022) were offered voluntary anti-HCV antibody testing.

A previous pilot study including adult individuals who attended the opening night parties after the COVID-19 lockdown in Sitges (Barcelona, Spain) on May 20th, 2021 was conducted to assess the acceptability of this strategy. Participants had been recruited at a point of care for SARS-CoV-2 testing before the party and had been scheduled for a compulsory re-test 5 days after the party (May 26th, 2021), during which they were offered voluntary anti-HCV antibody self-testing. Patients who declined to participate were asked for the reason for their decision, although it was not recorded.

Both studies were conducted in collaboration with the Association Colors Sitges Link, Sitges City Hall, and the main organizers of the events. Tents were set up on the street for a few hours during the day (morning or afternoon) with the collaboration of previously trained volunteers. They were located on beach accesses, party venues, and concerts. The volunteers approached potential participants and informed and offered users the opportunity to undergo a self-testing with a quick HCV saliva test, ensuring confidentiality with alphanumeric codes.

At the tent, participants provided their phone number, signed the test consent form, and completed a questionnaire on risk practices (only in the large-scale screening) and prior knowledge of hepatitis C. Finally, participants underwent the self-testing using the saliva test. The written informed consent form was available in Spanish, Catalan, and English. Once the test was completed, participants were informed that if the test was positive, they would be called within 20 min for confirmation testing of viremia in a blood drop (XPERT). Finally, if viremia was positive, attempts were made to link them to the healthcare system for hepatitis C treatment.

This study was carried out according to the ethical principles of the Helsinki Declaration (Fortaleza, Brazil) and the European (Law 2016/679) and local regulations (Organic Law 03/2018 for Personal Data Protection and Digital Rights). The study protocol for the large-scale screening was approved by the ethics committee of the Bellvitge University Hospital (L’Hospitalet de Llobregat, Spain).

### 2.2 Study objectives and assessments

The primary objective of this study was to determine the acceptability and feasibility of mass screening conducted on the street, measured by the number of participants. In the pilot study, the participation rate was calculated as the percentage of individuals who accepted to participate in the HCV screening who had performed the first SARS-CoV-2 testing before the party. The presence of anti-HCV antibodies (Ig G) was assessed using the OraQuick® HCV test on a saliva sample. This quick test has a 97.8% (95% confidence interval [CI], 93.2–99.4%) sensitivity and 100% (95% CI, 98.4–100%) specificity (gold standard: IgG antibody test for HCV by immunoassay [serum]) (19); and yields results in 20 min. Participants with positive anti-HCV results were subsequently offered a test for the presence of the HCV virus using the Xpert HCV Fingerstick®, which detects the HCV RNA on small blood samples (i.e., blood drop) with a lower detection limit of 100 UI/mL.

Secondary objectives were to determine the characteristics of the population included and the prevalence of HCV infection in this population (i.e., MSM). In addition to basic demographic characteristics, including age and nationality, variables considered were the number of participants with a previous HCV infection, the test for identification of risk practices (TIRP) results, and the self-perceived risk of sexually transmitted diseases (STDs) (Supplementary materials). In the pilot study, the question regarding the HCV serological status was “Do you know if you have had Hepatitis C”? (Yes, No, and Unknown), which was rephrased to “Do you have, or have you ever had Hepatitis C”? (Yes, No, and Unknown) for the large-scale screening. The TIRP is an 8-item questionnaire based on the European MSM Internet Survey and has been previously used to evaluate the relationships between certain risk practices and HCV infection (20, 21). The questionnaire is included in the protocol for HCV detection in MSM and transgender individuals developed by the Department of Health of the Government of Catalonia (22). The items have two possible answers (Yes: 1 point, and No: 0 points), with possible scores ranging from 0 to 8 points (Supplementary Table S1). A score ≥ 1 is considered a risk for STDs. Participants answered “What is your self-perceived risk of STDs?” on a 0 to 10 visual analog scale (VAS), where 0 was no risk, and 10 was maximum risk. These data were collected during a brief interview as participants and study personnel waited for the quick saliva test results.

### 2.3 Statistical analysis

Descriptive data were presented as the mean and standard deviation (SD), range (min, max), or interquartile range (IQR: 25th, 75th quartiles) for quantitative variables, and as frequencies and percentages for categorical variables. The HCV infection prevalence rate was calculated as the percentage of individuals with positive test results over those who participated.

This descriptive study aimed to assess the acceptability and feasibility of a large-scale HCV screening among at-risk communities; consequently, a formal sample size calculation was unnecessary. Nevertheless, 5000 MSM have attended the most popular events for this community in Sitges in previous years, and therefore, we anticipated sufficient potential participants would be available.

## 3 Results

### 3.1 Acceptability (screened participants) and HCV prevalence

A total of 1249 adults participated in the large-scale screening, of which 1197 (95.8%) were identified as MSM. Although the number of event attendees who declined to participate and their reasons for not participating could not be recorded, there was a common impression among the surveyors that the main reason individuals declined was due to self-reporting inclusion in a PrEP program, which involves regular STD screenings at 3–6 month intervals. In the pilot study, of the 337 individuals undergoing the post-party SARS-CoV-2 test, 216 agreed to be tested for anti-HCV antibodies, resulting in a 64.1% acceptability rate.

In the large-scale study, participants were screened at a speed of approximately 39 individuals/h, and in the pilot study, the screening for HCV was performed in 6 hours (approximately 36 individuals/h).

Four of the 1249 participants in the mass screening tested positive for anti-HCV antibodies, resulting in an HCV seroprevalence of 0.32%; all had undetectable levels of HCV RNA, and three had undetermined results (reading errors). None of the 216 participants in the pilot study tested positive for anti-HCV antibodies (210 were negative and six had undetermined results).

Participants in the large-scale screening were from 65 different nationalities: 35.05% were Spanish, and 90.91% were European, although all continents were represented in the study (Table 1). Participants had a median (IQR) age of 44 (35, 54) years. Additional characteristics of participants are summarized in Table 1. Participants in the pilot study had a median (IQR) age of 37.0 (23.0, 50.0) years, and 122 (56.2%) were male.

**Table 1:** Characteristics of participants in the large-scale screening.

Gender	
Men	1197 (95.8)
Age (years), *n* = 1197	
Mean (SD)	44.55 (12.84)
Median (IQR)	44.0 (35.0, 54.0)
Country of origin, *n* = 1236	
Europe	1000 (80.91)
Spain	433 (35.03)
United Kingdom	158 (12.78)
France	96 (7.77)
Italy	59 (4.77)
Germany	41 (4.13)
Netherlands	39 (3.16)
Ireland	37 (2.99)
Belgium	20 (1.62)
Switzerland	15 (1.21)
Hungary	9 (0.73)
Poland	8 (0.65)
Portugal	8 (0.65)
Austria	8 (0.65)
Czech Republic	8 (0.65)
Rumania	7 (0.57)
Grece	7 (0.57)
Sweden	7 (0.57)
Scotland	6 (0.49)
Estonia	5 (0.40)
Russia	4 (0.32)
Ukraine	4 (0.32)
Turkey	4 (0.32)
Cyprus	3 (0.24)
Armenia	3 (0.24)
Other European countries^a^	12 (0.97)
America	152 (12.3)
USA	53 (4.29)
Argentina	20 (1.62)
Venezuela	14 (1.13)
Brazil	12 (0.97)
Colombia	12 (0.97)
Canada	11 (0.89)
Mexico	7 (0.57)
Chile	5 (0.40)
Peru	4 (0.32)
Honduras	4 (0.32)
Bolivia	3 (0.24)
Ecuador	3 (0.26)
Other American countries^b^	4 (0.32)
Asia	33 (2.67)
Lebanon	4 (0.32)
Israel	4 (0.32)
China	3 (0.24)
Saudi Arabia	3 (0.24)
Other Asian Countries^c^	6 (0.48)
Oceania	19 (1.54)
Australia	18 (1.46)
New Zealand	1 (0.08)
Africa	6 (0.49)
Morrocco	3 (0.24)
Other African countries^d^	3 (0.24)
Other^e^	26 (2.1)

*N = 1249.*

IQR, interquartile range; SD, standard deviation.

^aAndorra, Luxemburg, Lithuania, Serbia: n = 2, 0.16%; Denmark, Iceland, Norway, Croatia: n = 1, 0.08%.^

^bGuatemala, Costa Rica, Cuba, Uruguay: n = 1, 0.08%.^

^cPhilippines, India, Japan, Taiwan, Pakistan, Siria: n = 1, 0.08%; Dubai: n = 2, 0.16%.^

^dArgelia, Kenya, South Africa: n = 1, 0.08%.^

^eMissreported.^

### 3.2 Awareness of serological HCV status and STD risk

**Figure 1:**
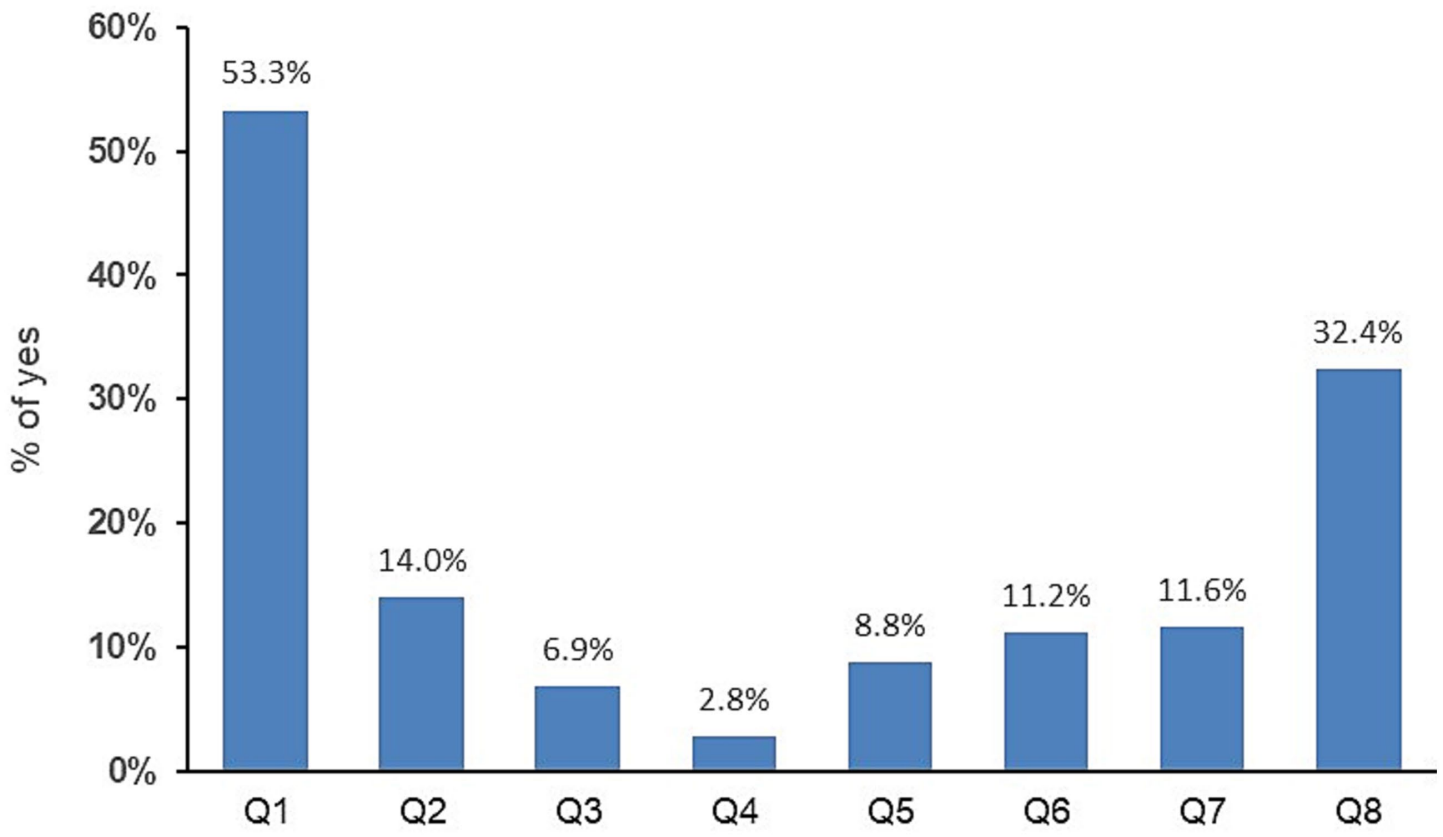
Percentage of Yes answers to the eight questions of the test for identification of risk practices.

Of the MSM in the large-scale study (*n* = 1197), 13% reported being unaware of their serological HCV status. A total of 1185 returned the TIRP questionnaire. The mean (SD) TIRP score was 1.40 (1.44) (*n* = 1062, data was invalid for 123 participants who returned partially completed questionnaires) (Table 2), with Question 1 (“Have you had receptive anal sex without a condom in the past 6 months?”) having the highest rate of Yes answers (53.30%), followed by Question 8 (“Are you using PrEP regularly?”) (32.39%) (Figure 1). Most participants (67.41%) reported some risk (*n* = 716 with TIRP scores ≥1), and one third (*n* = 346, 32.58%) reported no risk (i.e., TIRP score of 0). Regarding self-perceived STD risk, the mean (SD) score was 3.0 (2.82) (*n* = 969; data was missing for 180 participants) (Table 2).

**Table 2:** Self-perceived risk of sexually transmitted disease and test for identification of risk practices (TIRP) scores in the MSM community participating in the large-scale screening, *n* = 1197.

	*n*	Mean (SD)	Median (IQR)
Self-perceived risk	1041	3.0 (2.82)	2.0 (1.0, 5.0)
TIRP score	1062	1.4 (1.44)	1.0 (0.0, 2.0)

IQR, interquartile range; MSM, men who have sex with men; TIRP, test for identification of risk practices; SD, standard deviation.

In the pilot study, two (0.09%) participants reported previous HCV infection, 94 (43.3%) had no HCV infection, and 121 (55.8%) did not answer the question.

## 4 Discussion

This cross-sectional community-based study showed that the point-of-care approach on the street in massive events using quick oral tests allowed us to screen between 36 and 39 individuals per hour and reach many people from a community at risk. The prevalence of HCV antibodies among MSM (0.32%) was very low and even lower among individuals attending opening night parties (0%). Furthermore, our study confirmed that most MSM (67.41%) were at STD risk.

This study assessed a novel HCV screening strategy based on points of care at massive events. We found that the strategy was feasible and well accepted─64.1% acceptability rate in the pilot study─, reaching many individuals, likely due to the approach of offering a quick, non-invasive self-test on the street. A recent systematic review and meta-analysis showed that self-testing using quick tests on saliva samples was well-accepted (23), further supporting its use. Furthermore, the test yielded a negligible number of reading errors. In previous years, 3000–5000 individuals were estimated to attend the three main LGTB+ events in Sitges, and therefore, the number of participants in this study (*n* = 1249) was significant, considering the context of leisure and party. Thus, this novel strategy based on point-of-care tents on the street enabled data collection from a large sample in a short period, with very similar screening speeds in the pilot and large-screening study. This screening strategy may be implemented in the MSM community and may be a feasible option to screen other high-risk populations for HCV, such as homeless individuals and injected drug users, who are frequently outside routine care, likely due to increased mental health issues (4). Similarly, a recent study using a venue-based sampling strategy showed the feasibility of screening at-risk populations by approaching them at gathering venues (24). The success of setting up point-of-care tents on the street warrants further studies targeting other high-risk populations by setting them up at relevant locations.

The WHO developed a strategy for eliminating viral hepatitis as a public health threat by 2030 (2). Among different actions, this strategy included increasing the rate of detected infected individuals from 20% in 2015 to 30% in 2020 and 90% in 2030 (2). In 2017, Spain was one of the 12 countries on track to meet the WHO goals toward eradicating HCV by 2030 (25), thanks to removing treatment restrictions (26). Nevertheless, the Strategic Plan for Tackling Hepatitis C in the Spanish National Health System included screening of populations at risk (27). Likewise, the Department of Health of Catalonia, in its objectives of the Plan for the Prevention and Control of Hepatitis C in Catalonia, included the detection of unknown infections (28). Thus, screening and detecting HCV-infected individuals is a priority, and strategies reaching at-risk populations, such as the one presented in this study, should be considered. Importantly, this novel strategy carried out by different associations did not require appointments, social workers, or specific health care personnel, and, therefore, it is a cheap strategy that does not represent a burden on the health care system.

The prevalence of HCV antibodies among the 1466 individuals screened was lower than reported in other settings. The general prevalence of HCV varies among countries (29) and among MSM, the reported prevalences also differ. Previous studies reported a prevalence of 0.3–1.5% among HIV-negative MSM in different countries in Europe, Asia, and the Americas (3, 30). Recent systematic reviews and meta-analyses estimated pooled prevalences of 3.4 (95% CI: 2.8–−4.0) (31) and 5.9% (95% CI: 5.1–6.8) (29) regardless of HIV status. The estimated pooled prevalence in Spain was 6.3% (95% CI: 3.7–9.4) (29). The few studies assessing the seroprevalence of HCV in MSM in Catalonia estimated prevalences of 0.75% (95% CI: 0.1–1.6%) (32), 2.0% (95% CI: 0.7–4.8) (33) and, more recently, 0.52% (95% CI: 0.33–0.82) (34), in line with our study. In this regard, the MSM included in the large screening study were from 65 different nationalities and may not necessarily reflect the prevalence in Spain. Nevertheless, the low prevalence found in this study among MSM, similar to that in the no-MSM community, may be explained by the awareness of STDs and the importance of healthcare, with frequent blood tests, in this community.

In addition, the recruitment strategy and populations analyzed may further explain differences in the seroprevalences reported in our setting. In this regard, HCV prevalence is higher in MSM who inject drugs and those who are HIV-positive (29, 31). Although most of the previous studies were conducted in community care centers and STD clinics, the reasons for consultation may differ, and the MSM populations screened may have different characteristics regarding the prevalence of HIV infection, use of injected drugs, and other factors influencing HCV transmission (32–34). Our study included all adults attending popular events in the MSM community and, in the pilot study, adult men and women attending opening night parties, regardless of HIV status and injected drug use, capturing general prevalences irrespective of other factors. Despite reporting exposure to STD and sexual risk behaviors, the HCV seroprevalence in MSM participating in the screening was low. Recent studies have reported that the incidence of HCV infection among MSM using PrEP is increasing, suggesting an association between PrEP use and sexual risk behaviors in MSM (3). Furthermore, the sexualized use of drugs (Chem sex) is associated with risk behaviors (35, 36), in addition to the intrinsic risk of sharing injecting equipment. Moreover, a trend towars sexual risk behaviors among MSM in high-income countries has been reported (37). These observations indicate that MSM are at high risk for HCV transmission, and therefore, screenings in this community should still be a priority.

The results from this study should be interpreted in the context of limitations associated with the screening method. Regarding the HCV RNA negativity in all four HCV AB positive individuals, which gave us a positive predictive value of 0, this may be related to the considerably low prevalence of the infection in the study population; however, we cannot exclude the possibility of a false negative result for the HCV RNA test. On the other hand, although the quick oral test enabled the identification of anti-HCV antibodies outside the clinic, seroconversion may take a long time, precluding the detection of acute infection (38) which could result in a false-negative outcome in some of the study participants. Although we were able to collect information regarding the sexual behaviors of the screened population, we did not collect data regarding HIV infection, PreP use, or injected drug use, among other factors related to HCV transmission, precluding subgroup analyses. However, the association between these factors and HCV transmission has been reported, and the general goal of our study was to provide evidence of the feasibility of a novel point-of-care self-testing strategy on the street to reach populations at risk. Given the nature and purpose of this study, data was collected on paper, limiting the number of variables collected and their quality. Moreover, no selection strategy was implemented other than setting the point of care during MSM events, so we cannot ensure that our sample is representative of the study population. Finally, while our study focused on demonstrating the feasibility of screening, we must acknowledge that the detection yield was notably low, as no participants with active infection were identified. This outcome may be attributed to a selection bias, including the participation of individuals who had previously undergone hepatitis C testing or those who were at a low risk for this infection. Additionally, it could reflect a genuine decrease in the prevalence of hepatitis C in this population due to current antiviral treatments, or it may result from false negative findings, as previously discussed. Nevertheless, the primary message we aim to convey in this study is that this approach represents a highly cost-effective strategy in terms of economic resources and time. We believe it would be worthwhile to apply this screening model in populations with a higher prevalence of hepatitis C and limited access to healthcare services.

## 5 Conclusion

A point-of-care strategy on the street using quick oral anti-HCV antibodies self-testing at massive MSM events is feasible, well-accepted, quick, and cheap, facilitating the screening of many individuals of communities at risk in a short period. This strategy may be useful to perform massive screening at the community and reach other populations at risk of HCV infection.

## Data Availability

The data analyzed in this study is subject to the following licenses/restrictions: the datasets for this study are available from the corresponding author upon reasonable request. Requests to access these datasets should be directed to sonia.albertos@gmail.com.

## References

[1] World Health Organization (WHO). Hepatitis C Key Facts. (2023). Available at: https://www.who.int/news-room/fact-sheets/detail/hepatitis-c (Accessed January 20, 2024).

[2] WHO. Global Health sector strategy on viral hepatitis 2016–2021. (2016). Available at: http://apps.who.int/iris/bitstream/10665/246177/1/WHO-HIV-2016.06-eng.pdf (Accessed January 20, 2024).

[3] Nijmeijer BM Koopsen J Schinkel J Prins M Geijtenbeek TB. Sexually transmitted hepatitis C virus infections: current trends, and recent advances in understanding the spread in men who have sex with men. J Int AIDS Soc. (2019) 22:e25348. doi: 10.1002/jia2.25348 31468692 PMC6715947

[4] Stasi C Silvestri C Voller F. Update on hepatitis C epidemiology: unaware and untreated infected population could be the key to elimination. SN Compr Clin Med. (2020) 2:2808–15. doi: 10.1007/s42399-020-00588-3, PMID: 33103061 PMC7568689

[5] Terrault N. Sexual activity as a risk factor for hepatitis C. Hepatology. (2002) 36:S99–S105. doi: 10.1002/hep.1840360713 12407582

[6] Centers for Disease Control and Prevention (CDC), MMWR. (2011). Sexual Transmission of Hepatitis C Virus Among HIV-Infected Men Who Have Sex with Men --- New York City, 2005--2010. Available at: https://www.cdc.gov/mmwr/preview/mmwrhtml/mm6028a2.htm (Accessed May 5, 2024). 21775948

[7] van de Laar TJW van der Bij AK Prins M Bruisten SM Brinkman K Ruys TA. Increase in HCV incidence among men who have sex with men in Amsterdam most likely caused by sexual transmission. J Infect Dis. (2007) 196:230–8. doi: 10.1086/518796, PMID: 17570110

[8] Cotte L Cua E Reynes J Raffi F Rey D Delobel P. Hepatitis C virus incidence in HIV-infected and in preexposure prophylaxis (PrEP)-using men having sex with men. Liver Int Off J Int Assoc Study Liver. (2018) 38:1736–40. doi: 10.1111/liv.13922 29959866

[9] Volk JE Marcus JL Phengrasamy T Hare CB. Incident hepatitis C virus infections among users of HIV Preexposure prophylaxis in a clinical practice setting. Clin Infect Dis Off Publ Infect Dis Soc Am. (2015) 60:1728–9. doi: 10.1093/cid/civ129, PMID: 25694649 PMC4850931

[10] Fierer DS Schinkel J. Redefining the paradigm: the role of sexual networks and sexualized drug use in the transmission of hepatitis C virus among men who have sex with men. J Infect Dis. (2023) 228:657–61. doi: 10.1093/infdis/jiad265, PMID: 37486348

[11] Folch C Fernández-Dávila P Ferrer L Soriano R Díez M Casabona J. High prevalence of drug consumption and sexual risk behaviors in men who have sex with men. Med Clin (Barc). (2015) 145:102–7. doi: 10.1016/j.medcli.2014.04.030, PMID: 25256434

[12] World Health Organization (WHO). WHO Global Hepatitis Report. (2017). Available at: https://www.who.int/publications/i/item/9789241565455 (Accessed May 5, 2024).

[13] Petroff D Wolffram I Bätz O Jedrysiak K Kramer J Berg T. Confirmation of guideline-defined hepatitis C screening strategies within the “check-Up35+” examination in the primary care setting. Liver Int. (2023) 43:785–93. doi: 10.1111/liv.15516 36621849

[14] Houri I Horowitz N Katchman H Weksler Y Miller O Deutsch L. Emergency department targeted screening for hepatitis C does not improve linkage to care. World J Gastroenterol. (2020) 26:4878–88. doi: 10.3748/wjg.v26.i32.4878, PMID: 32921964 PMC7459203

[15] Eisen L Mor Z Madar M Rabinovitch R Dadon Y Sheffer R. Hepatitis C virus elimination program among prison inmates, Israel. Emerg Infect Dis. (2023) 29:2358–61. doi: 10.3201/eid2911.230728, PMID: 37877627 PMC10617329

[16] Stämpfli D Imfeld-Isenegger TL Hersberger KE Messerli M. Hepatitis C virus screening in community pharmacies: results on feasibility from a Swiss pilot. BMC Infect Dis. (2023) 23:384. doi: 10.1186/s12879-023-08362-1, PMID: 37286975 PMC10246867

[17] Waked I Esmat G Elsharkawy A El-Serafy M Abdel-Razek W Ghalab R. Screening and treatment program to eliminate hepatitis C in Egypt. N Engl J Med. (2020) 382:1166–74. doi: 10.1056/NEJMsr1912628 32187475

[18] Owens D Davidson K Krist A Barry M Cabana M Caughey A. Screening for hepatitis C virus infection in adolescents and adults: US preventive services task force recommendation statement. JAMA. (2020) 323:970–5. doi: 10.1001/jama.2020.1123, PMID: 32119076

[19] Cha YJ Park Q Kang ES Yoo BC Park KU Kim JW. Performance evaluation of the OraQuick hepatitis C virus rapid antibody test. Ann Lab Med. (2013) 33:184–9. doi: 10.3343/alm.2013.33.3.184, PMID: 23667844 PMC3646192

[20] Fernández-Dávila P Folch C Ferrer L Soriano R Diez M Casabona J. Hepatitis C virus infection and its relationship to certain sexual practices in men-who-have-sex-with-men in Spain: results from the European MSM internet survey (EMIS). Enferm Infecc Microbiol Clin. (2015) 33:303–10. doi: 10.1016/j.eimc.2014.07.012, PMID: 25444047

[21] The EMIS Network. The European Men-Who-Have-Sex-With-Men Internet Surey (EMIS). (2024) Available at: https://www.emis-project.eu/

[22] Generalitat de Catalunya. (2018), Available at: https://salutpublica.gencat.cat/web/.content/minisite/aspcat/vigilancia_salut_publica/vih-sida-its/04_Hepatitis_viriques/guies_i_protocols/Protocol-HSH-i-persones-Trans.pdf

[23] Perazzo H Castro R Villela-Nogueira C Torres M Silva SL Cardoso SW. Acceptability and usability of oral fluid HCV self-testing for hepatitis C diagnosis: a systematic review and meta-analysis. J Viral Hepat. (2023) 30:838–47. doi: 10.1111/jvh.13876, PMID: 37485619

[24] Vaux S Chevaliez S Saboni L Sauvage C Sommen C Barin F. Prevalence of hepatitis C infection, screening and associated factors among men who have sex with men attending gay venues: a cross-sectional survey (PREVAGAY), France, 2015. BMC Infect Dis. (2019) 19:315. doi: 10.1186/s12879-019-3945-z, PMID: 30971207 PMC6458747

[25] Kåberg M Weiland O. Hepatitis C elimination - Macro-elimination. Liver Int Off J Int Assoc Study Liver. (2020) 40:61–6. doi: 10.1111/liv.14352 32077600

[26] CDA Foundation. Just 12 countries worldwide on track to eliminate hepatitis C infection by 2030, with United Kingdom, Italy and Spain among those joining the list. (2024) Available at: https://cdafound.org/just-12-countries-worldwide-on-track-to-eliminate-hepatitis-c-infection-by-2030-with-united-kingdom-italy-and-spain-among-those-joining-the-list/

[27] Spanish Ministry of Health. Strategic Plan For Tackling Hepatitis C In The Spanish National Health System. (2015). Available at: https://www.sanidad.gob.es/ciudadanos/enfLesiones/enfTransmisibles/hepatitisC/PlanEstrategicoHEPATITISC/docs/PEAHC_eng.pdf

[28] Department of Health. Generalitat de Catalunya. Pla de prevenció i control de l’Hepatitis C a Catalunya. (2018). Available at: https://salutpublica.gencat.cat/ca/ambits/vigilancia_salut_publica/vih-sida-its-hv/Hepatitis-viriques/descripcio/

[29] Zheng Y Ying M Zhou Y Lin Y Ren J Wu J. Global burden and changing trend of hepatitis C virus infection in HIV-positive and HIV-negative MSM: a systematic review and Meta-analysis. Front Med. (2021) 8:774793. doi: 10.3389/fmed.2021.774793, PMID: 34966758 PMC8710739

[30] Silva V Kerr L Kendall C Mota R Guimarães M Leal A. Hepatitis C virus prevalence among men who have sex with men: a cross-sectional study in 12 Brazilian cities. BMC Infect Dis. (2023) 23:705. doi: 10.1186/s12879-023-08690-2 37858036 PMC10588169

[31] Jin F Dore G Matthews G Luhmann N Macdonald V Bajis S. Prevalence and incidence of hepatitis C virus infection in men who have sex with men: a systematic review and meta-analysis. Lancet Gastroenterol Hepatol. (2021) 6:39–56. doi: 10.1016/S2468-1253(20)30303-4, PMID: 33217341

[32] Saludes V Folch C Morales-Carmona A Ferrer L Fernàndez-López L Muñoz R. Community-based screening of hepatitis C with a one-step RNA detection algorithm from dried-blood spots: analysis of key populations in Barcelona, Spain. J Viral Hepat. (2018) 25:236–44. doi: 10.1111/jvh.12809, PMID: 29053912

[33] Coll J Videla S Leon A Ornelas A García F Fernández E. Early detection of HIV infection and of asymptomatic sexually transmitted infections among men who have sex with men. Clin Microbiol Infect. (2018) 24:540–5. doi: 10.1016/j.cmi.2017.08.012 28843621

[34] Palma D Alarcón M, García de Olalla P Guerras JM Pericas C García J. Hepatitis C antibody prevalence and active hepatitis C infection in HIV-negative gay, bisexual, and other men who have sex with men in Barcelona and Madrid, Spain (march 2018-march 2021). IJID Reg (2023);8:95–104, doi: 10.1016/j.ijregi.2023.07.001, PMID: 37554356 PMC10404990

[35] Santos GM Coffin PO Das M Matheson T DeMicco E Raiford JL. Dose-response associations between number and frequency of substance use and high-risk sexual behaviors among HIV-negative substance-using men who have sex with men (SUMSM) in San Francisco. J Acquir Immune Defic Syndr. (2013) 63:540–4. doi: 10.1097/QAI.0b013e318293f10b 23572012 PMC4671496

[36] Celentano DD Valleroy LA Sifakis F MacKellar DA Hylton J Thiede H. Associations between substance use and sexual risk among very young men who have sex with men. Sex Transm Dis. (2006) 33:265–71. doi: 10.1097/01.olq.0000187207.10992.4e 16434886

[37] Hess KL Crepaz N Rose C Purcell D Paz-Bailey G. Trends in sexual behavior among men who have sex with men (MSM) in high-income countries, 1990-2013: a systematic review. AIDS Behav. (2017) 21:2811–34. doi: 10.1007/s10461-017-1799-1, PMID: 28555317 PMC5708163

[38] European Association for the Study of the Liver. EASL recommendations on treatment of hepatitis C 2018. J Hepatol. (2018) 69:461–511. doi: 10.1016/j.jhep.2018.03.026 29650333

