# Supplementary material for: A large-scale screening of hepatitis C among men who have sex with men in the community using saliva point-of-care testing
Source: Front Public Health. 2024 Dec 9;12:1478195. doi: 10.3389/fpubh.2024.1478195 (PMC11663926; doi:10.3389/fpubh.2024.1478195)
Supplement: Supplementary file 1 [file Table_1.docx]

**Supplementary Materials for the Submission Entitled “A Large-Scale Screening of Hepatitis C Among Men Who Have Sex With Men in the Community Using Saliva Point-of-Care Testing” by Albertos et al.**

**Supplementary Methods**

**TEST FOR IDENTIFICATION OF RISK PRACTICES (tirp)**

| **Do you have or have you ever had hepatitis C?**  **(Yes/No/Unknown).** |  |
| --- | --- |

From 0 to 10 (0 being No risk and 10, Maximum risk. **Mark with an X your probability of having a sexual transmitted disease (STD) .**

| **0** | **1** | **2** | **3** | **4** | **5** | **6** | **7** | **8** | **9** | **10** |
| --- | --- | --- | --- | --- | --- | --- | --- | --- | --- | --- |
| **0 10**  **No risk Maximum risk** | | | | | | | | | | |

|  |  |  |  |
| --- | --- | --- | --- |
| **1** | **Have you had receptive anal sex without a condom in the past 6 months?*** | **YES** | **NO** |
|  |  |  |  |
| **2** | **Have you shared sex toys in the last 6 months?*** | **YES** | **NO** |
|  |  |  |  |
| **3** | **Have you practiced fisting without gloves in the past 6 months?*** | **YES** | **NO** |
|  |  |  |  |
| **4** | **Have you injected drugs in the past year?*** | **YES** | **NO** |
|  |  |  |  |
| **5** | **Have you shared "curls" (instruments for intranasal drug administration) to snort drugs in the past 12 months?*** | **YES** | **NO** |
|  |  |  |  |
| **6** | **Have you had any sexually transmitted ulcerative infections (syphilis, herpes simplex virus) during the past 12 months?*** | **YES** | **NO** |
|  |  |  |  |
| **7** | **Have you practiced ChemSex in the last year?*** | **YES** | **NO** |
|  |  |  |  |
| **8** | **Are you using PrEP regularly?*** | **YES** | **NO** |
|  |  |  |  |
